# The Histone H3 Lysine 9 Methyltransferase DIM-5 Modifies Chromatin at *frequency* and Represses Light-Activated Gene Expression

**DOI:** 10.1534/g3.114.015446

**Published:** 2014-11-25

**Authors:** Catherine E. Ruesch, Mukund Ramakrishnan, Jinhee Park, Na Li, Hin S. Chong, Riasat Zaman, Tammy M. Joska, William J. Belden

**Affiliations:** Department of Animal Sciences, Rutgers, The State University of New Jersey, School of Environmental and Biological Sciences, New Brunswick, New Jersey 08901

**Keywords:** circadian rhythm, chromatin, DNA methylation, heterochromatin

## Abstract

The transcriptional program controlling the circadian rhythm requires coordinated regulation of chromatin. Characterization of the chromodomain helicase DNA-binding enzyme CHD1 revealed DNA methylation in the promoter of the central clock gene *frequency* (*frq*) in *Neurospora crassa*. In this report, we show that the DNA methylation at *frq* is not only dependent on the DNA methyltransferase DIM-2 but also on the H3K9 methyltransferase DIM-5 and HP1. Histone H3 lysine 9 trimethylation (H3K9me3) occurs at *frq* and is most prominent 30 min after light-activated expression. Strains lacking *dim-5* have an increase in light-induced transcription, and more White Collar-2 is found associated with the *frq* promoter. Consistent with the notion that DNA methylation assists in establishing the proper circadian phase, loss of H3K9 methylation results in a phase advance suggesting it delays the onset of *frq* expression. The *dim-5* deletion strain displays an increase in circadian-regulated conidia formation on race tubes and there is a synthetic genetic interaction between *dim-5* and *ras-1^bd^*. These results indicate DIM-5 has a regulatory role in muting circadian output. Overall, the data support a model where facultative heterochromatic at *frq* serves to establish the appropriate phase, mute the light response, and repress circadian output.

The circadian clock controls endogenous, self-sustained, and temperature-compensated oscillations in gene expression. The eukaryotic and metazoan circadian rhythm, present in most but not all organisms, requires chromatin remodeling and modifications for proper regulation because the core mechanism underlying all eukaryotic clocks is a transcriptional negative feedback loop. In *Neurospora crassa*, the circadian negative feedback loop consists of the positive transcriptional activators WHITE COLLAR (WC) -1 and WC-2 that form the white collar complex (WCC), and the negative elements are FREQUENCY (FRQ) and FRQ-interacting RNA helicase (FRH) (Bell-Pedersen *et al.* 2005; Dunlap *et al.* 2007; Heintzen and Liu, 2007; Brunner and Kaldi 2008). The WCC controls expression of *frq*, and FRQ:FRH abrogates *frq* expression (Aronson *et al.* 1994; Crosthwaite *et al.* 1995; Crosthwaite *et al.* 1997; Cheng *et al.* 2005). Posttranslation modifications to FRQ, WC-1, WC-2, and histones occur during the course of the day and in response to light, and they serve to help regulate the coordinated timing of activation, repression, and turnover (Garceau *et al.* 1997; Schwerdtfeger and Linden, 2000; Dunlap *et al.* 2007; Baker *et al.* 2009; Tang *et al.* 2009). There is a significant amount of literature documenting the molecular mechanics of WCC-mediated activation (Ballario *et al.* 1998; Talora *et al.* 1999; Froehlich *et al.* 2002; He *et al.* 2002), FRQ phosphorylation, and turnover (He *et al.* 2003, 2005, 2006; Baker *et al.* 2009, Tang *et al.* 2009, Querfurth *et al.* 2011), but far less is known regarding the role of chromatin in modulating both activation and inhibition of the circadian transcriptional cycle. In addition, the molecular mechanisms of phase determination are still largely undefined.

Chromatin remodeling and posttranslational histone modifications are essential for proper regulation of *frq*, and these help establish either permissive or nonpermissive states for transcription in a phase-dependent manner. Chromatin-associated negative feedback repression is brought about by the histone H3 lysine 4 methyltransferase SET1 and the ATP-dependent remodeling enzyme Clockswitch (Belden *et al.* 2007b; Raduwan *et al.* 2013). Clockswitch remodels chromatin at the nucleosome distal to the C-box to generate an inaccessible state for WC-2 binding. Other ATP-dependent chromatin-remodeling enzymes include SWI/SNF and Clock ATPase. Both SWI/SNF and Clock ATPase facilitate *frq* expression by generating a rhythm in nucleosome occupancy at the C-box (Cha *et al.* 2013; Wang *et al.* 2014). Other activation- and elongation-dependent modifications include histone H3 acetylation and H2B ubiquitination (Grimaldi *et al.* 2006; Belden *et al.* 2007b). Activation and/or elongation also appear to require the chromodomain-helicase DNA-binding remodeling enzyme CHD1, although the role of CHD1 in this process remains enigmatic (Belden *et al.* 2011). Loss of CHD1 results in an increase in DNA methylation in *frq* and normal DNA methylation is dependent on the DNA methyltransferase DIM-2 (Defective In Methylation-2) (Belden *et al.* 2011). Further characterization of DNA methylation at *frq* revealed that it requires a functional clock, the *frq* natural antisense transcript *qrf*, and is entirely dependent on WCC-mediated transcription (Belden *et al.* 2011).

The overwhelming majority of the studies on DNA methylation in *Neurospora* have been performed at relics of repeat-induced point mutations (RIP’d regions), repetitive regions that have been mutated and packaged into heterochromatin. In *Neurospora*, all DNA methylation requires the DNA methyltransferase DIM-2 (Kouzminova and Selker 2001). DNA methylation also requires the histone H3 lysine 9 methyltransferase DIM-5 (KMT1) (Tamaru and Selker, 2001, Tamaru *et al.* 2003), and heterochromatin protein 1 (HP1) (Freitag *et al.* 2004), along with additional subunits that are part of the DIM-5 protein complex, DCDC (DIM-5/-7/-9, CUL4/DDB1 Complex) (Lewis *et al.* 2010). The molecular mechanism of heterochromatin formation at RIP’d regions involves recruitment of DCDC components by DIM-7/DIM-5 leading to H3K9me3. HP1 then binds H3K9me2/3 via its chromodomain and recruits DIM-2 (Honda and Selker 2008). In addition to methylation at repetitive regions, there is also methylation at convergent transcripts. However, methylation at convergent transcripts is relatively undefined, especially at the level of chromatin, but appears to require noncoding RNA. These convergent transcripts, which include the *frq* locus, give rise to dicer-independent, small interfering RNA (disiRNA) (Lewis *et al.* 2009, Dang *et al.* 2013). The role of DIM-5 or HP1 in DNA methylation at disiRNA loci is still unresolved. Unlike DNA methylation at RIP’d regions, H3K9me3 is reported to be dependent on DNA methylation at convergent transcripts, suggesting that DNA methylation either precedes H3K9me3 or H3K9me3 is rapidly removed if DNA methylation is absent (Dang *et al.* 2013). Moreover, the role of DIM-5 in DNA methylation and how it affects expression at convergent loci has not been examined. Loss of DNA methylation at *frq* has only minor effects on clock function and manifests as a small phase advance (Belden *et al.* 2011). Collectively, this finding suggests DNA methylation may serve as a terminal modification that plays an ancillary role relative to other underlying factors needed to establish DNA methylation, like H3K9me3 or HP1 binding.

In this report, we show that both DIM-5 and HP1 are required for DNA methylation at *frq*. Genetic analysis indicates that *Δdim-5* can suppress the hypermethylation phenotype in *Δchd1*, which further solidifies the requirement for H3K9 methylation and HP1 for DNA methylation at *frq* in *Neurospora*. H3K9me3 occurs at *frq* and is established in response to light. H3K9me3 is prominently present at the light to dark transition and loss of H3K9 methylation results in a phase advance. In addition, DIM-5 is needed to inhibit light-mediated expression of a subset of light-activated genes examined and this coincides with a peak in H3K9me3 that occurs 30 min after exposure to light. These data support a model for a diurnal rhythm in facultative heterochromatin.

## Materials and Methods

### Strains and growth conditions

A complete list of strains used in this study is presented in Supporting Information, Table S1. The wild-type strain, Fungal Genetics Stock Center (FGSC) 2489, and a clock wild type (WT) containing *ras-1^bd^* (XB136-6) were used as the parent strains in all crosses. The *hpo* strain was kindly provided by Dr. Eric Selker. The starting ∆*dim-5* strain (FGSC15885) was generated by the *Neurospora* knockout consortium and obtained from the FGSC. Standard crosses were used to obtain the ∆*dim-5*;∆*chd-1* and ∆*dim-2*; ∆*chd-1*, and these were genotyped by polymerase chain reaction (PCR). Established culture conditions were used for all circadian and light experiments. Liquid media consisted of 1× Vogel’s, 0.17% arginine, 2% glucose, and supplemented with an additional 50 μg/mL biotin. Race tube media contained 1× Vogel’s, 0.17% arginine, 0.1% glucose, and 50 μg/mL biotin. Luciferase rhythms were detected by crossing ∆*dim-5* to a strain with the FRQ-Luciferase translational fusion (Larrondo *et al.* 2012), and images were captured with an Evolve EMCCD camera and then analyzed with μManager software (Edelstein *et al.* 2010).

### DNA methylation and chromatin immunoprecipitation (ChIP)

DNA methylation at *frq* was performed as previously described (Belden *et al.* 2011). The ChIP experiments were modified slightly to improve the H3K9me3 ChIP. Briefly, tissue was crosslinked in 1% formaldehyde for 15 min and then quenched with 0.125 M glycine for an additional 15 min. Tissue was lysed by grinding in the presence of liquid nitrogen then suspended in 10 mL of FA lysis buffer (0.05M Hepes, pH 7.4; 0.15 M NaCl; 0.001 M Ethylenediaminetetraacetate (EDTA) acid; 1% TX-100; 0.1% sodium dodecyl sulfate [SDS]) containing protease inhibitors (PIs; 2.0 μg/mL leupeptin, 2.0 μg/mL pepstatin A, 1.0 mM phenylmethanesulfonylfluoride). The lysates were sonicated 2 times at 25% power on a Misonix ultrasonic disruptor to provide additional cell lysis and then the lysates were spun at 2000× *g* to remove cellular debris. The cleared lysates were then spun at 100,000 × *g* to obtain chromatin-enriched pellets. The pellet was suspended in 10 mL of fresh FA lysis buffer + PI and then sonicated 6 times at 65% to an average size of 500 bp. Equal amounts of sheared chromatin was immunoprecipitated with WC-2 (Denault *et al.* 2001) or H3K9me3 (Millipore) antibodies. Oligonucleotides used in the quantitative PCR have been described previously (Belden *et al.* 2007b). Relative enrichment for a subset of the experiments was determined by subtracting the background then normalizing the values relative to values obtained at the *wc-2* locus.

### RNA extraction and real-time PCR

RNA was extracted from ground frozen tissue under RNase-free conditions using Trizol as previously described (Raduwan *et al.* 2013). For reverse-transcription (RT)-PCR, 5.0 μg of purified RNA was treated with Turbo DNaseI following manufacturer’s guidelines. Equal concentrations of DNaseI treated RNA was used in a reverse transcription reaction using High Capacity cDNA Reverse Transcription Kit (Applied Biosystems) with a random hexamer. Quantitative PCR products were detected with SYBR green. Oligonucleotides for *frq*, *wc-1*, and *vvd* are reported elsewhere (Raduwan *et al.* 2013). In all RT-PCR experiments, the relative value was normalized to the *rac-1* mRNA.

### Protein analysis

Total soluble protein was extracted from the ground frozen tissue by suspending a portion of the tissue in 200 μL of protein extraction buffer plus PI. The lysates were spun for 10 min at 16,000 × *g* to remove cell debris. An equal concentration of total protein was diluted in sample buffer (25% glycerol; 0.125 M Tris-HCl, pH 6.8; 4% SDS; 5% 2-mercaptoethanol), heat denatured, resolved on a 6.5% SDS polyacrylamide gel, transferred to polyvinylidene difluoride membranes, and detected with antibodies specific to FRQ (Garceau *et al.* 1997) and WC-1 (Lee *et al.* 2000).

## Results

### DNA methylation at the *frq* promoter requires both DIM-5 and HP1

**Figure 1:**
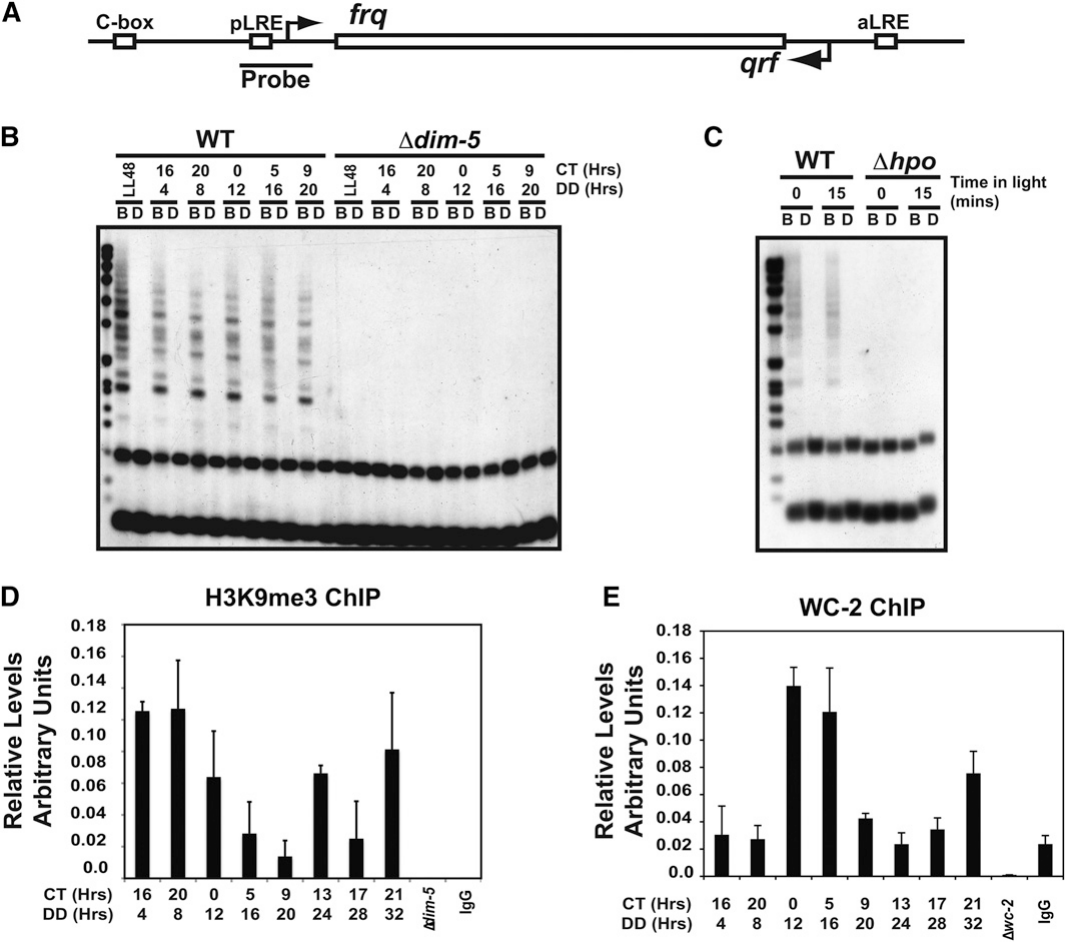
DNA methylation at *frq* requires both DIM-5 and heterochromatin protein 1. (A) A schematic representation of the *frq* gene. The probe used for the Southern blots marks the region at which the methylation is greatest. (B) DNA methylation at *frq* was assayed by performing methylation sensitive Southern blots. Total genomic DNA was isolated from WT (FGSC2489) and *∆dim-5* (XB18-11) grown under circadian conditions and harvested at the indicated times in the dark (DD) corresponding to a given circadian time (CT). The DNA was digested with the methyl-sensitive *Bfu*CI (labeled B) and methyl-insensitive *Dpn*II (labeled D) restriction endonucleases. A probe corresponding to a region of the *frq* promoter was used to detect methylated DNA. (C) Same as in A except the *hpo* strain was used. (D) H3K9me3 ChIP was performed on wild-type chromatin after growth under circadian conditions. Oligonucleotides used in the quantitative polymerase chain reaction were specific a region around the pLRE/TSS. Values are normalized relative to the signal obtained using oligonucleotides specific for *wc-2*. (E) Same as in D except the ChIP was performed with an antibody specific to WC-2 and oligonucleotides were specific for the C-box. The error bars represent the standard error of the mean. ChIP, chromatin immunoprecipitation; DIM, Defective In Methylation.

We recently demonstrated that DNA methylation occurs in the clock gene *frq* and that the methylation was dependent on the DNA methyltransferase DIM-2 (Belden *et al.* 2011). At RIP’d regions, recruitment of DIM-2 requires association with HP1 and DIM-5-mediated H3K9me3 (Tamaru and Selker, 2001; Tamaru *et al.* 2003; Freitag *et al.* 2004; Honda and Selker 2008). Therefore, we sought to examine the requirement of DIM-5 for DNA methylation at *frq*. We performed methylation sensitive Southern blots on strains lacking DIM-5 compared to WT (FGSC 2489) grown under circadian conditions (Figure 1A). Genomic DNA was isolated and digested in side-by-side reactions with the methyl-sensitive restriction enzyme *Bfu*CI compared with the methyl-insensitive isoschizomer *Dpn*II then probed with a region specific to the proximal light−regulated element (pLRE) contained in the *frq* promoter. As a control for complete DNA digestion, we examined a region of mitochondrial DNA that is in excess relative to genomic DNA but is never methylated (Figure S1A). We were unable to detect any partially digested DNA in the *Bfu*CI samples from *Δdim-5*, indicating that DIM-5 and H3K9 methylation are necessary for DNA methylation at *frq*.

Next, we examined whether HP1 was likewise involved in DNA methylation at *frq*. As with *Δdim-5*, we performed a DNA methylation sensitive Southern blot comparing the *hpo* mutant to WT at two time points. In *hpo*, the *frq* promoter is devoid of all detectable DNA methylation (Figure 1B), indicating that both DIM-5 and HP1 are required for DNA methylation at *frq*. As with *Δdim-5*, we confirmed complete digestion by examining a region of the mitochondrial genome (Figure S1B).

### H3K9me3 at the *frq* promoter

To ascertain whether DNA methylation is directly dependent on H3K9me3 at *frq* and rule out indirect consequences that might arise in ∆*dim-5* or *hpo* strains, we performed ChIP with an antibody specific to H3K9me3 using both *Δdim-5* and a non-specific IgG as controls. We tested whether H3K9me3 was present at regions in *frq* over time. H3K9me3 was detected at a variety of locations throughout *frq*, but was most prominently present at regions near the transcriptional start site (TSS; the region containing DNA methylation). Background subtracted and normalized ChIP samples fluctuated over circadian time, but H3K9me3 consistently appears highest at the light to dark transition (DD4/CT16-DD8/CT20) (Figure 1D). To validate H3K9me3 at *frq*, we also performed the H3K9me3 ChIP on isolated nuclei and found H3K9me3 levels above *Δdim-5* (Figure S2A). As a positive control for H3K9me3 ChIP, we used oligonucleotides specific to a subtelomeric region that is known to have H3K9me3 (Figure S2B) (Smith *et al.* 2008). These results indicate that H3K9me3 occurs at *frq* and is dependent on DIM-5. Cumulatively, we performed the H3K9me3 ChIP over 12 times using 3 slightly different ChIP methodologies and rarely observed a circadian rhythm. Instead, H3K9me3 appeared to have noncircadian fluctuations but was universally elevated relative to *Δdim-5*. The reason for this is unclear but is consistent with the dynamic DNA methylation at *frq* that is present, but has a tendency to fluctuate and dissipate with time in the dark. Importantly, the maximum levels of DNA methylation (in the dark) and the underlying H3K9me3 occur at times shortly after the light to dark transition (Belden *et al.* 2011). Moreover, the peak in H3K9me3 occurs at transcriptional transitions in *frq* expression from highly expressed to repressed (*e.g.*, light to dark transition), implying that DIM-5 is likely involved in muting *frq* expression via facultative heterochromatin formation. The lack of a robust circadian rhythm in H3K9me3 was not due to errors in processing the ChIP lysates because there is a high-amplitude circadian rhythm in WC-2 binding to the C-box using the identical samples (Figure 1E). Instead, it is likely that complex regulation of *frq*, which includes the expression of a natural antisense transcript *qrf* (Kramer *et al.* 2003), leads to complex regulation of H3K9me3. Furthermore, subsequent analyses of DIM-5 and H3K9me3 at *frq* (see next section) demonstrate it is not needed for oscillations under circadian conditions, so the lack of a robust circadian rhythm in H3K9me3 is inconsequential.

### DIM-5 controls phasing and represses circadian output

**Figure 2:**
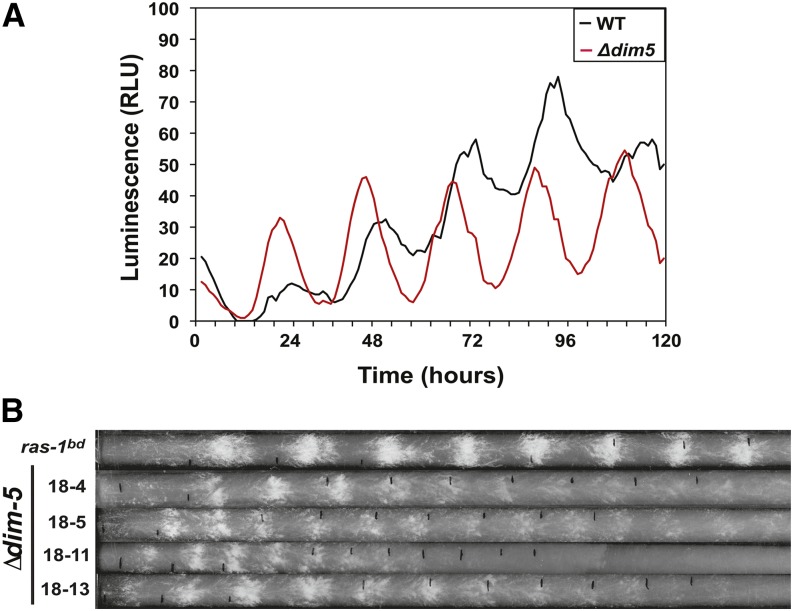
DIM-5 is required for circadian phase and suppresses circadian output. (A) A *frq-luciferase* translation fusion was used to monitor rhythms in *frq* for 5 d in *Δdim-5* (XB151-2) compared with an isogenic WT (XL92-12) sampling every hour. (B) Four independent *Δdim-5* strains (X18-4, X18-5, X18-11, and X18-13) were compared with *ras-1^bd^* (XB136-6) on race tubes to examine circadian output.

To explore the requirement for DIM-5 and H3K9 methylation in clock function, we crossed ∆*dim-5* to a strain containing the *frq* luciferase translation fusion construct. We observed rhythms in *frq-luc* that had a period consistent with rhythmic expression (~22 hr). However, there was a significant phase advance of ~4 hr that was greater in magnitude than what was previously observed for *dim-2* mutants (~2 hr for ∆*dim-2*) (Figure 2A) (Belden *et al.* 2011). We next examined whether there were any defects in circadian output. We crossed ∆*dim-5* to a strain carrying the *ras-1^bd^* allele to monitor rhythms in conidia formation on race tubes. Unfortunately, we were unable to isolate a strain harboring both *ras-1^bd^* and ∆*dim-5* from a standard cross even after repeated attempts. We screened 80 *dim-5*::*hph* spores for the *ras-1^bd^* allele and were unable to isolate a single strain containing both alleles. This was quite surprising because *ras-1* and *dim-5* are separated by 1.6 Mb on Linkage Group IV and while mapping *ras-1^bd^*, we determined that 1 cM was approximately equal to 30 kb in regions surrounding *ras-1* (Belden *et al.* 2007a). This finding suggested a synthetic genetic effect between RAS-1 signaling and H3K9me3 that we examined further (Figure S3). Regardless, in routine phenotype testing for *ras-1^bd^*, we noticed that spores containing ∆*dim-5* had elevated circadian conidia formation independent of *ras-1^bd^* and exhibited near circadian periodicity (Figure 2B). To confirm that loss of heterochromatin caused heightened circadian output independent of *ras-1^bd^*, we crossed the ∆*dim-5* strain to WT (FGSC 2489) and observed an identical phenotype. Collectively these data suggest that DIM-5 and RAS-1 function in parallel pathways and mediate the amplitude of clock controlled gene (*ccg*) expression as determined by elevated circadian output. Consistent with the luciferase data, we noted a small phase advance and only a minor change in period length, although the linear growth rate of ∆*dim-5* was much slower than that of WT or *ras-1^bd^* (Figure 2B). This finding supports the possibility that DIM-5 may be a global regulator controlling the amplitude of *ccg* gene expression. However, we cannot rule out pleiotropic effects from a lack of silencing in repetitive regions or chromosome segregation defects known to occur in ∆*dim-5* (Lewis *et al.* 2010).

To further understand *dim-5*−dependent defects in the clock, we examined relative levels of *frq* transcript in *∆dim-5* compared with WT (FGSC2489) under circadian conditions by RT-PCR. Surprisingly, we were unable to detect any significant differences in *frq* expression (Figure S4A); a result that we confirmed by Western blot of FRQ (Figure S4B). The lack of significant differences in expression likely arises due to the nature of the phenotype, variability in pipeting, and sampling at 4-hr intervals that collectively make it difficult to detect subtle defects. In addition, we examined whether loss of HP1 altered *frq* expression and like *∆dim-5*, we observed no major changes in the *hpo* strain (Figure S5). These data indicate that like DNA methylation, neither H3K9me3 nor HP1 was necessary for rhythmic *frq* expression in constant darkness, but loss of H3K9me3 does cause a phase defect suggesting an issue with the light response.

### DIM-5 is required to attenuate the light response

**Figure 3:**
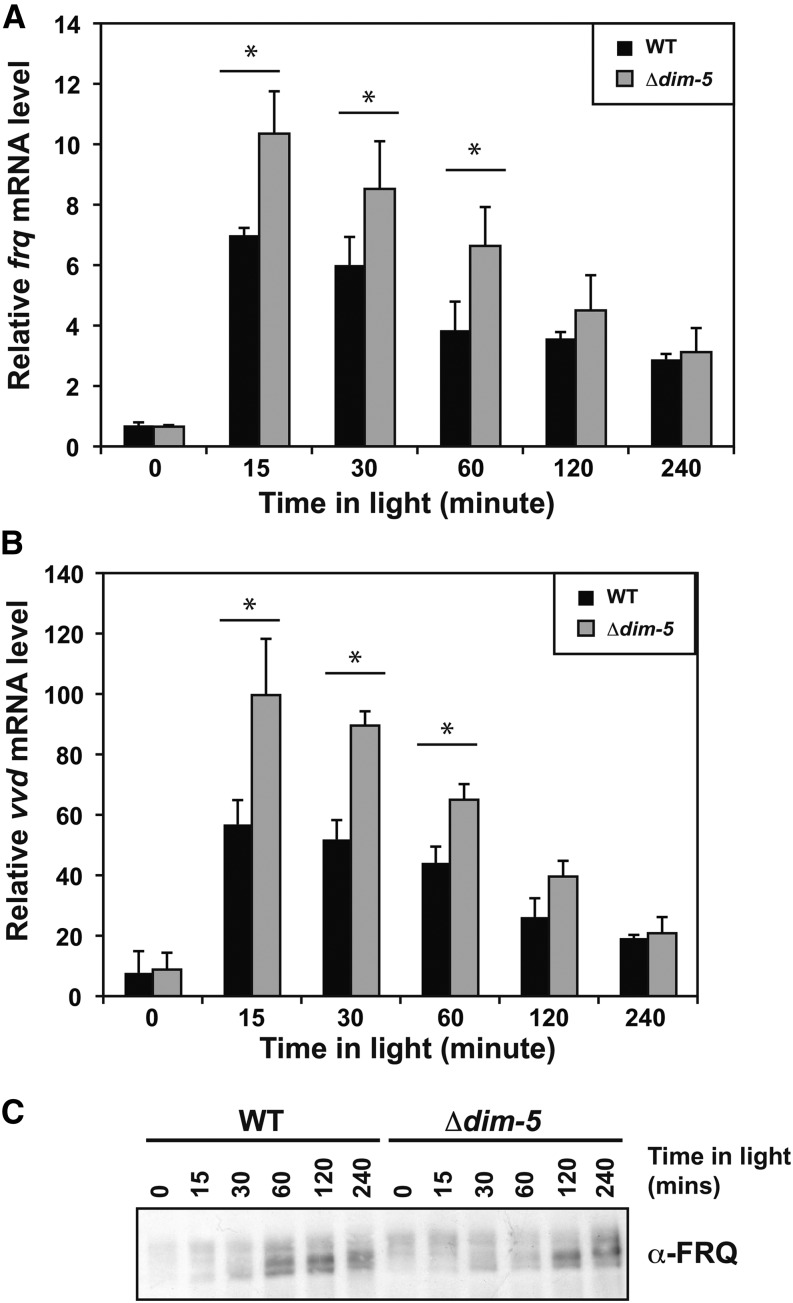
DIM-5 is a corepressor of light-mediated expression. Wild-type (WT; FGSC2489) and *Δdim-5* (XB18-11) were grown in the dark then transferred to saturating light and harvested at the indicated times. RNA was extracted and transcript abundance was measured by reverse-transcription polymerase chain reaction with oligonucleotides specific for (A) *frq* and (B) *vvd*. The data are averages of four experiments, and the error bars represent the SEM. Asterisks highlight significant differences using a *P* < 0.05. (C) Representative western blot showing FRQ protein abundance. DIM, Defective In Methylation; FRQ, FREQUENCY.

The phase advance and heightened output suggests that H3K9me3 may be more important under diurnal light-dark cycles. This idea is consistent with a requirement for circadian entrainment to maintain DNA methylation (Belden *et al.* 2011). In addition, *frq* and *qrf* are both light induced and this sense/antisense expression is responsible for disiRNA production that is needed for DNA methylation (Kramer *et al.* 2003, Lee *et al.* 2010). Therefore, we sought to determine whether H3K9me3 was involved in the *Neurospora* light response. To examine this, we grew WT and *∆dim-5* strains in the dark for 40 hr to remove residual light effects. Then cultures were exposed to saturating light conditions and harvested at defined times. We observed an increase in both *frq* (Figure 3A) and *vvd* (Figure 3B) in response to light at a subset of time points in *∆dim-5* compared to WT, but we were unable to see any significant differences in *wc-1* (Figure S6). The changes in *frq* expression did not significantly alter the level of FRQ protein under identical conditions suggesting there may be an increase in abortive transcripts, a defect in processing mature *frq* mRNA, an increase in *qrf*, or a delay in translation. Regardless, we routinely observed elevated levels of FRQ in constant light suggesting that differences in protein levels can be detected after prolonged periods in the light (Figure S4B).

### H3K9me3 is established in response to light

**Figure 4:**
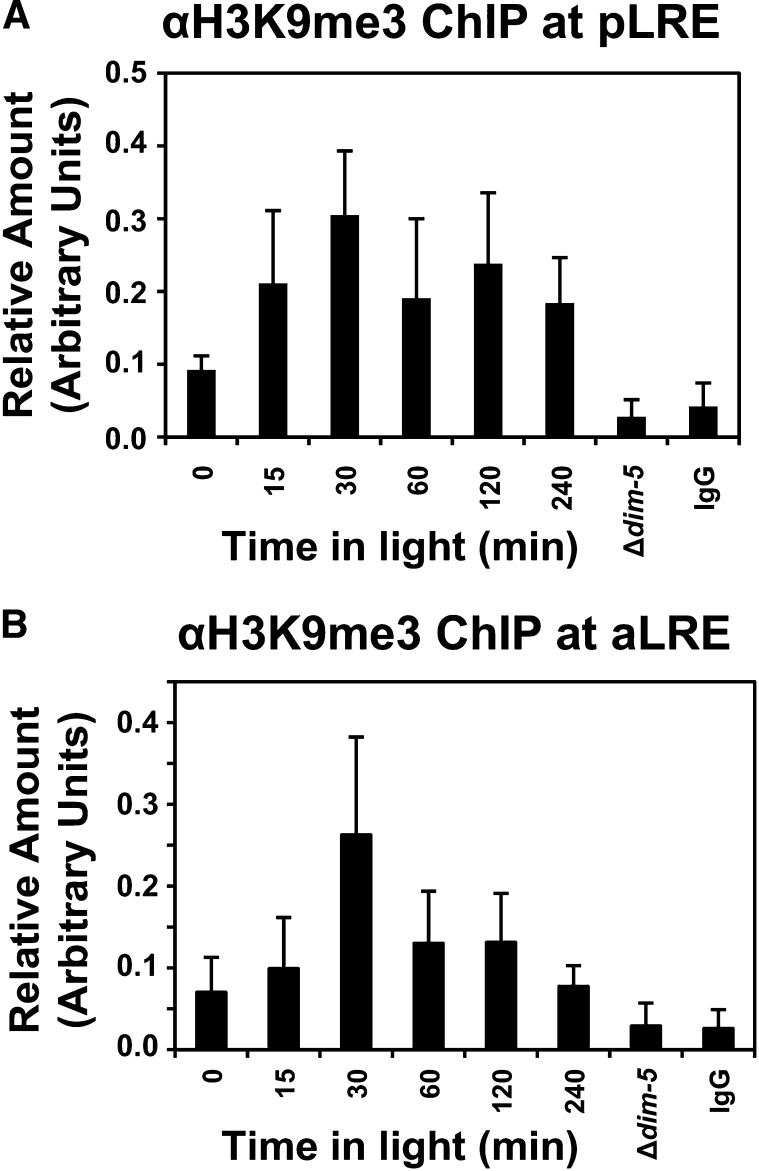
H3K9me3 is established at *frq* in response to light. The levels of H3K9me3 at *frq* were measured by ChIP under light-inducing conditions. WT (FGSC2489) was grown in the dark for 40 hr then transferred to saturating light and crosslinked at the indicated times prior to processing. An antibody specific to H3K9me3 was used to ascertain the level of H3K9me3 at *frq*. Relative levels of H3K9me3 were determined by quantitative polymerase chain reaction of ChIP DNA using oligonucleotides specific to the (A) pLRE and (B) aLRE. Both *Δdim-5* and a nonspecific IgG were used as controls. The data are averages of 4 experiments and the error bars represent the standard error of the mean. The asterisks indicate significant differences using a *P* < 0.05. ChIP, chromatin immunoprecipitation; pLRE, proximal light−regulated element. aLRE, antisense light-regulated element.

The defect in *frq* and *vvd* repression in the light suggested that H3K9me3 is presumably established upon exposure to light, an idea consistent with *frq/qrf* generating disiRNAs needed for DNA methylation (Dang *et al.* 2013). Therefore, we proceeded to examine the kinetics of H3K9me3 at *frq* in response to light by performing H3K9me3 ChIP. The levels of H3K9me3 at pLRE peaked 30 min after light exposure, a time when repressive modifications would be expected (Figure 4A). In addition, we also observed H3K9me3 at the aLRE. The amount of H3K9me3 at the aLRE (antisense light-regulated element in response to light was lower than that observed for the pLRE (Figure 4B), which is consistent with the relative ratio of both transcripts. Combined, these data indicate that H3K9me3 occurs shortly after light exposure and is needed to reduce the amount of transcription originating from *frq*. It is important to note that the pLRE is adjacent to the TSS and using either TSS or pLRE oligonucloetides gives nearly identical results. Thus, H3K9me3 occurs near the pLRE/TSS in response to light.

### WC-2 binding is enhanced in the absence of H3K9me3

**Figure 5:**
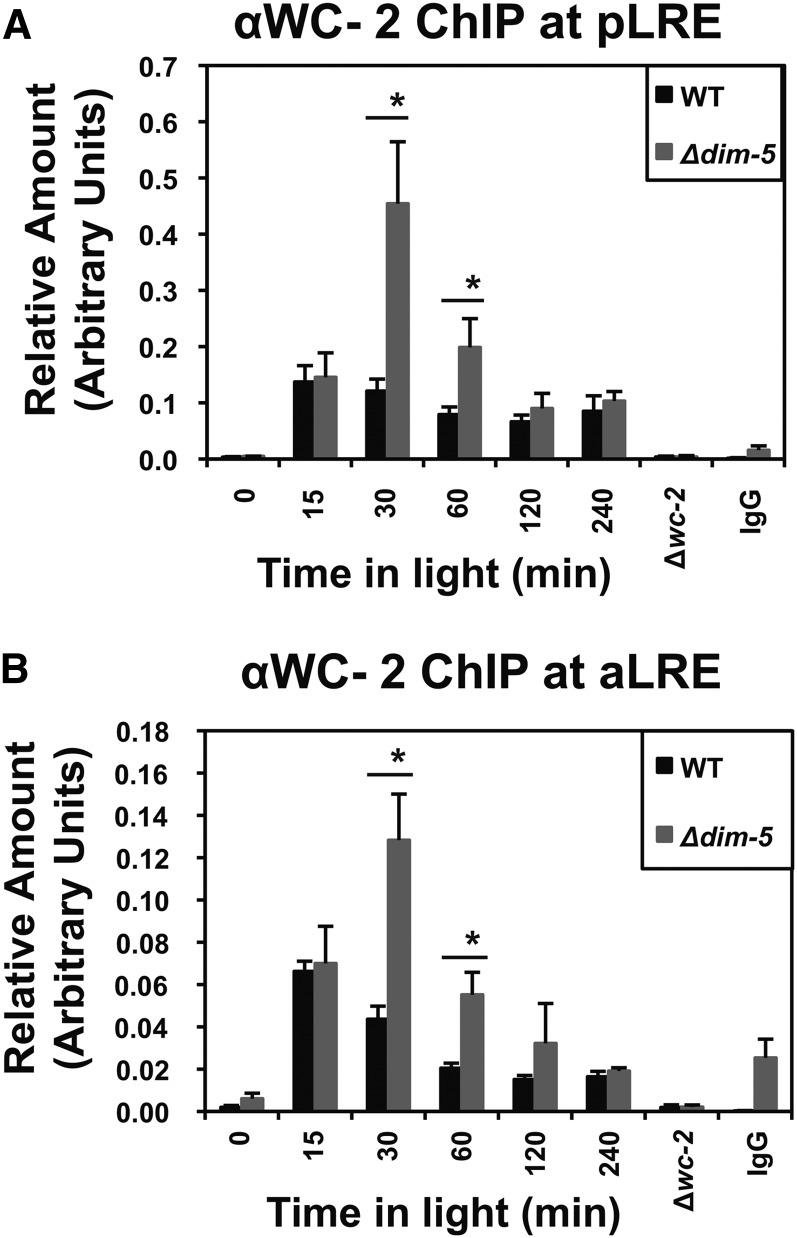
H3K9me3 inhibits WC-2 binding. WC-2 binding at the (A) PLRE and (B) aLRE promoter elements was determined by ChIP of DNA from WT (FGSC2489) and *Δdim-5* strains exposed to light for the indicated times. *Δwc-2* and a nonspecific IgG were used as controls. The data are averages of four independent experiments and the asterisk indicates times at which greater levels of WC-2 are found associated with the pLRE or aLRE. The error bars represent the SEM with *P* < 0.05. ChIP, chromatin immunoprecipitation; pLRE, proximal light−regulated element. aLRE, antisense light-regulated element.

The appearance of H3K9me3 at *frq* after exposure to light, and the increase in light-regulated gene expression, suggests that the levels of WCC associated with the *frq* promoter may be elevated in *∆dim-5*. To examine this, we performed a WC-2 ChIP in WT and *∆dim-5* under light-inducing conditions and examined binding at the pLRE (Figure 5A) and aLRE (Figure 5B). We observed a substantial increase in WC-2 association with both elements in *∆dim-5* relative to WT at the 30- and 60-minute time points. Collectively, these data suggest that the canonical role of H3K9me3 in creating a more condensed heterochromatic state is preserved.

### Chd1 hypermethylation is lost in Δ*dim-5*

As a final confirmation that DIM-5 was a key component needed for heterochromatin formation and DNA methylation at *frq*, we sought to determine whether loss of *dim-5* could suppress the hypermethylation phenotype observed in strains lacking *chd1*. We previously reported that loss of *chd1* caused more extensive DNA methylation compared with WT (Belden *et al.* 2011). Why this occurs is confounding, but if DNA methylation at *frq* is dependent on DIM-5, then DIM-5 should suppress the hypermethylation phenotype. To examine this, we crossed *∆dim-5* to *∆chd1* and obtained spores lacking both alleles. We next performed a methylation sensitive Southern blot examining the methylated region in *frq* promoter. As an added control, we also created a *∆dim-2*, *∆chd1* double mutant. Loss of either *dim-5* or *dim-2* suppressed the DNA hypermethylation typically observed in *chd1* solidifying the notion that both are required for DNA methylation at *frq* while inferring that H3K9me3 may likewise be elevated and sustained in *∆chd1*. We next explored any potential synthetic growth defects that arise in the double *∆dim-5*, *∆chd1* strain. It is clear from the slowed growth rate of *∆dim-5*, *∆chd1* double deletion that both DIM-5 and CHD1 have separable functions in opposing pathways.

## Discussion

Chromatin remodeling and histone modifications are essential for proper circadian control, and the number of enzymes and modifications that occur at *frq* continues to grow. In this report, we show that DIM-5−dependent H3K9me3 and HP1 are required for DNA methylation at *frq*. In addition *dim-5* can suppress the hypermethylation phenotype of *chd1*. Further characterization indicates that loss of H3K9 methylation results in a phase advance. The phase advance observed in *∆dim-5* is approximately 2 hr earlier than *∆dim-2*, indicating that H3K9me3 is more important for setting the phase than DNA methylation. We also demonstrate that H3K9me3 is established in response to light and loss of H3K9me3 results in elevated WCC binding to the *frq* promoter and an increase in light-activated *frq* expression. Thus, H3K9me3 appears to be involved in muting the high level of light-mediated expression and is likely more important in the diurnal expression that accompanies changes in environmental conditions. This finding is entirely consistent with the DNA methylation that requires circadian entrainment. The mechanisms involved in establishing H3K9me3 are still unknown but are likely established by coordinated transcription of *frq* and *qrf* that give rise to disiRNA. Although still not fully elucidated, it seems likely that facultative heterochromatin at *frq* is highly conserved and appears analogous to facultative heterochromatin formation in *S. pombe*. Facultative heterochromatin formation in *S. pombe* uses the exosome instead of RNA interference machinery to generate small, interfering RNA (siRNA) molecules from noncoding RNA and mRNA to direct the KMT1, Clr4 (The DIM-5 ortholog) (Zofall *et al.* 2012). Ergo, the mechanism used to direct H3K9me3 at *frq* likely shares some similarities to RNA interference−mediated heterochromatin formation, but the method used to generate the siRNA (in this case disiRNA) is different (Verdel and Moazed 2005).

The timing of H3K9me3 and the corresponding results on expression are consistent with a role in establishing diurnal facultative heterochromatin at *frq*. H3K9me3 appears to peak at times when light and circadian expression are normally attenuated and loss of *dim-5* in the light leads to elevated expression of both *frq* and *vvd*. This indicates that DIM-5 plays a repressive role in light-mediated expression at least at these two loci. Interestingly, *∆dim-5* has heightened conidia formation on race tubes relative to an isogenic WT strain, strongly suggesting it is necessary to control the diurnal amplitude of *ccg* expression even though DIM-5 is dispensable for rhythmic expression. This finding is quite surprising, because in standard culture conditions, with relatively high sugar concentration (2.0% *vs.* 0.1% on race tubes), there is a defect in conidia development in *dim-5* mutants (Tamaru and Selker 2001). However, this result is consistent with circadian cell culture experiments that demonstrate a rhythm in facultative heterochromatin formation at the *ccg* D-element Binding Protein (*Dbp*) (Ripperger and Schibler 2006). In addition, while completing this work, we determined that the human ortholog of DIM-5, Suppressor of variegation 39 (Suv39h), is a component of the Period complex and is needed for H3K9me2 and H3K9me3 at the *Per1* E-box (Duong and Weitz 2014). siRNA against *Suv39h1* caused a shorter period (Duong and Weitz 2014), indicating that H3K9me3 serves to inhibit the onset of CLOCK:BMAL1-initiated transcription.

DIM-5 plays a significant role in down regulating a subset of light-activated genes examined herein but only a supportive role in circadian *frq* expression via controlling circadian phase. These data suggest that H3K9me3 governs chromatin compaction at *frq* and provides a refractory period after the light to dark transition. Interestingly, it was recently reported that disiRNA gives rise to DNA methylation, but H3K9me3 is lost in the absence of DNA methylation (Dang *et al.* 2013). In light of those findings, and the work reported here, it seems likely that DNA methylation may prevent the recruitment of a histone H3 lysine 9 demethylase. It is tempting to speculate that CHD1 may be involved in recruiting this unidentified histone H3 lysine 9 demethylase.

**Figure 6:**
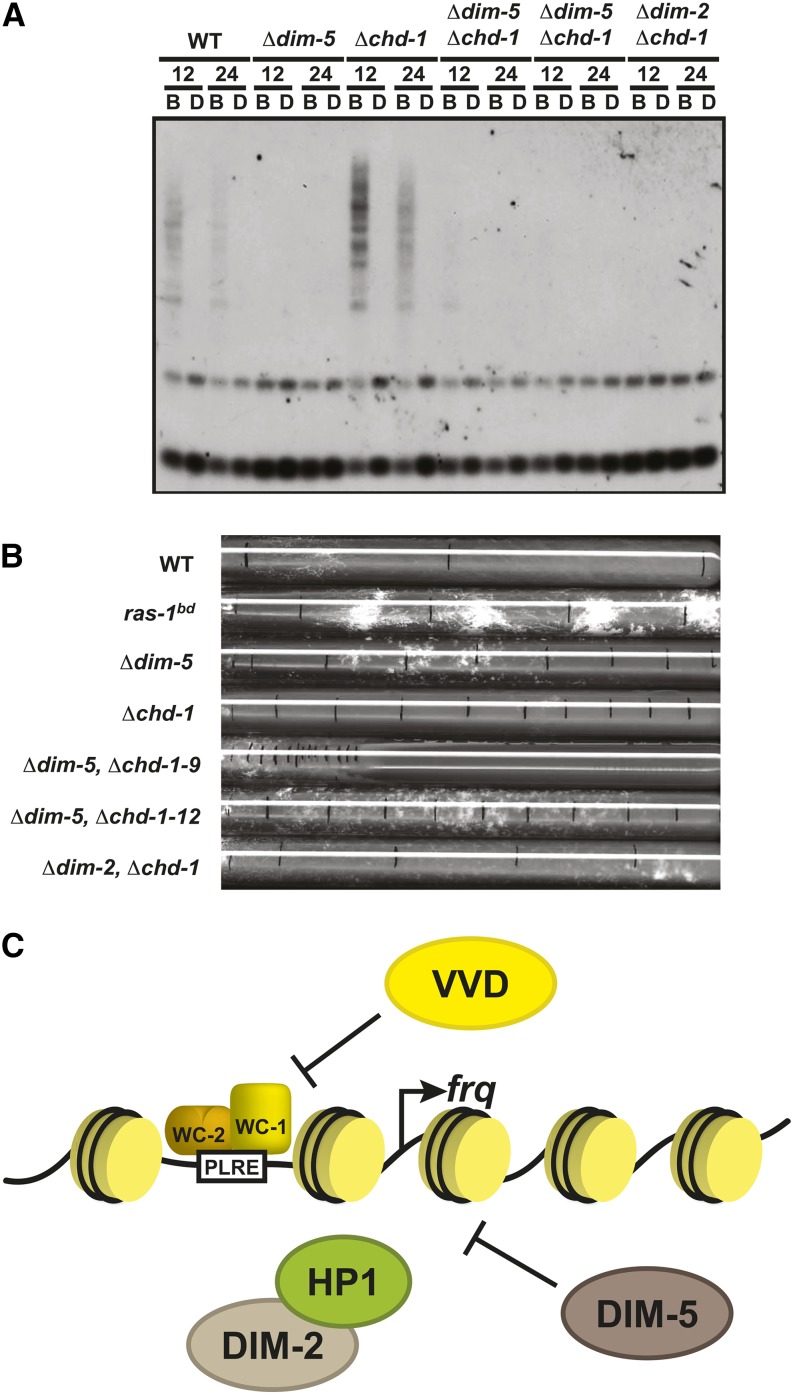
Loss of *dim-5* can suppress the *chd1* hypermethylation phenotype. (A) Methylation-sensitive Southern blot performed as described in Figure 1 comparing WT (FGSC2489), *Δdim-5* (XB18-11), *Δchd1* (XB136-6) and two separate isolates containing the double-deletion strains *Δdim-5;Δchd1* (XB230-9 and XB230-12) with *Δdim-2;Δchd1* (XB104-2) added as a control. (B) Growth rates of the *Δdim-5;Δchd1* double deletion indicate a genetic interaction that results in additive affect. (C) Schematic representation of DIM-5 function at *frq*. The diagram illustrates how DIM-5 and VVD function in separate, but parallel pathways to inhibit WCC-mediated expression of *frq*. DIM, Defective In Methylation; HP1, heterochromatin protein 1; VVD, WC, WHITE COLLAR; WT, wild type.

The mechanism of light-mediated heterochromatin formation at *frq* (or other light activated genes) is still largely undefined and represents an area that merits further study. This is especially true considering that adapted, light-activated genes are not completely silenced by heterochromatin. Instead they have an intermediate constitutive level of expression. Ergo, although H3K9me3 represses expression, it does not completely silence expression. This also raises the question of whether H3K9me3 is dependent on VIVD (VVD) or functions in a parallel pathway. On the surface, these may appear to be in a sequential biochemical pathway. However, simple logic indicates that this is highly unlikely and instead these function in a parallel pathway. Support for this comes from our previous work showing the *frq* promoter is methylated in strains lacking *vvd* (Belden *et al.* 2011). Because DNA methylation is dependent on H3K9 methylation, then H3K9me3 must be independent of VVD. Moreover, light adaptation is still present in *∆dim-5* indicating that *vvd*-mediated down regulation of the light response is still intact. A model consistent with the data are that VVD inhibits the WCC through direct interaction (Chen *et al.* 2010; Hunt *et al.* 2010; Malzahn *et al.* 2010) whereas DIM-5 establishes a more condensed chromatin state (Figure 6C).

Another interesting observation in this report is the notion that loss of H3K9 methylation has a synthetic effect with *ras-1^bd^* and that both *∆dim-5* and *ras-1^bd^* have somewhat-similar phenotypes on race tubes. Oncogenic *ras* mutations are present in 30–40% of all cancers, and thus inhibitors of KMT1 enzymes may represent ideal chemotherapeutics for a variety of carcinomas. Recently, it was found that overexpression of the KMT1, SUV39H1 in zebrafish can suppresses a rhabdomyosarcoma model produced by KRAS^G12D^ overexpression (Albacker *et al.* 2013). These observations indicate that the *Neurospora ras-1^bd^* strain may serve as an ideal model to screen for potential chemotherapeutic agents or used as a vehicle for a synthetic lethal screen.

## Supplementary Material

Supporting Information
