# Supplementary material for: The Histone H3 Lysine 9 Methyltransferase DIM-5 Modifies Chromatin at frequency and Represses Light-Activated Gene Expression
Source: G3 (Bethesda). 2014 Nov 25;5(1):93–101. doi: 10.1534/g3.114.015446 (PMC4291474; doi:10.1534/g3.114.015446)
Supplement: Supporting Information [file supp_5_1_93__index.html]

The Histone H3 Lysine 9 Methyltransferase DIM-5 Modifies Chromatin at frequency and Represses Light-Activated Gene Expression — Supporting Information 

# The Histone H3 Lysine 9 Methyltransferase DIM-5 Modifies Chromatin at *frequency* and Represses Light-Activated Gene Expression

## Supporting Information for Ruesch *et al.*, 2015

**Files in this Data Supplement:**

- Supporting Information - Table S1 and Figures S1-S6 (PDF, 542 KB)
- Table S1 - Strain table. (PDF, 379 KB)
- Figure S1 - Control for DNA methylation in *Δdim-5* and *hpo*. (PDF, 222 KB)
- Figure S2 - Supplemental H3K9me3 ChIP data. (PDF, 180 KB)
- Figure S3 - Synthetic affect between *Δdim-5* and *ras-1bd*. (PDF, 228 KB)
- Figure S4 - Molecular Rhythms in *Δdim-5*. (PDF, 222 KB)
- Figure S5 - Molecular Rhythms in *hpo*. (PDF, 207 KB)
- Figure S6 - DIM-5 is not required for *wc-1* expression. (PDF, 215 KB)
