# Supplementary material for: The Histone H3 Lysine 9 Methyltransferase DIM-5 Modifies Chromatin at frequency and Represses Light-Activated Gene Expression
Source: G3 (Bethesda). 2014 Nov 25;5(1):93–101. doi: 10.1534/g3.114.015446 (PMC4291474; doi:10.1534/g3.114.015446)
Supplement: Supporting Information [file supp_g3.114.015446_015446SI.pdf]

G3·Genes | Genomes | Genetics

The Histone H3 Lysine 9 methyltransferase DIM-5 modifies chromatin at *frequency* and represses light-activated gene expression

Catherine E. Ruesch, Mukund Ramakrishnan, Jinhee Park, Na Li, Hin S. Chong, Riasat Zaman, Tammy M. Joska and William J. Belden*

Department of Animal Sciences
Rutgers, The State University of New Jersey
School of Environmental and Biological Sciences
New Brunswick, NJ. 08901 USA

* Corresponding author: belden@aesop.rutgers.edu

DOI: 10.1534/g3.114.015446

Table S1

Strain Table

Strain	Genotype	Source
FGSC2489	OR74, A (WT)	FGSC
FGSC11124	*wc-2::hph*, a	FGSC
FGSC15885	[*dim-5::hph, a*]	FGSC
XB18-4	*dim-5::hph*	This Study
XB18-5	*dim-5::hph*	This Study
XB18-11	*dim-5::hph*	This Study
XB18-13	*dim-5::hph*	This Study
90-2	*mus51::bar, a*	Colot *et al.*, 2006
T101	[*dim-2::nat1; mus51::bar, a*]	This Study
XB100-9	*chd-1::hph, A*	This study
XB104-2	*dim-2::nat1; chd-1::hph*	This Study
XB131-6	*chd-1::hph*, a	Belden *et al.*, 2011
XB136-6	*ras-1^{bd}*, A	Raduwan *et al.*, 2013
XB142-8	*dim-5::hph*, A	This Study
XL94-10	*frq-luc::bar, a*	Larrondo *et al.*, 2012
XB151-2	*dim-5::hph; frq-luc::bar*	This Study
XB230-9	*dim-5::hph; chd-1::hph*	This Study
XB230-12	*dim-5::hph; chd-1::hph*	This Study

* FGSC denotes Fungal Genetics Stock Center

Figure S1.

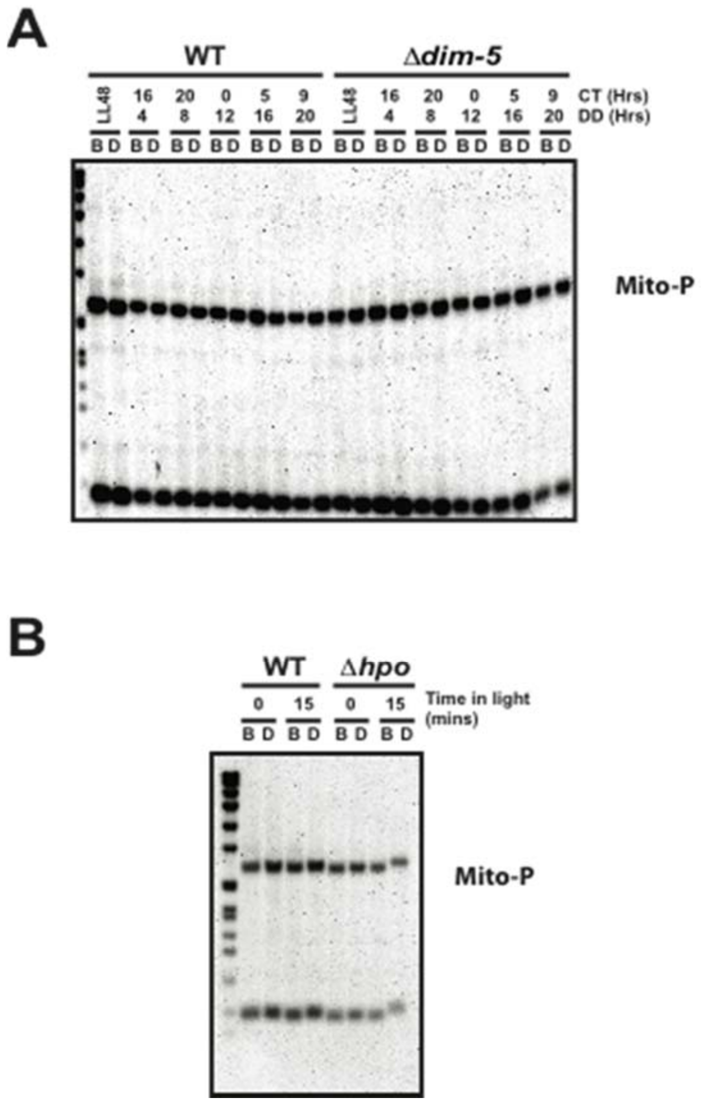

Figure S1 Control for DNA methylation in *Δdim-5* and *hpo*. (A) Methylation sensitive Southern blot from Figure 1 was stripped and re-probed with a probe that hybridizes to a region within the mitochondria genome. The figure indicates complete digestion in WT (FGSC2489) and *Δdim-5* (XB18-11). Time in the dark (DD) and corresponding circadian time (CT) is indicated. The restriction enzymes are the methyl-sensitive *Bfu*CI (B) and insensitive *Dpn*II (D). (B) Same as in A except the membrane was from Figure 1B that examined the *hpo* strain.

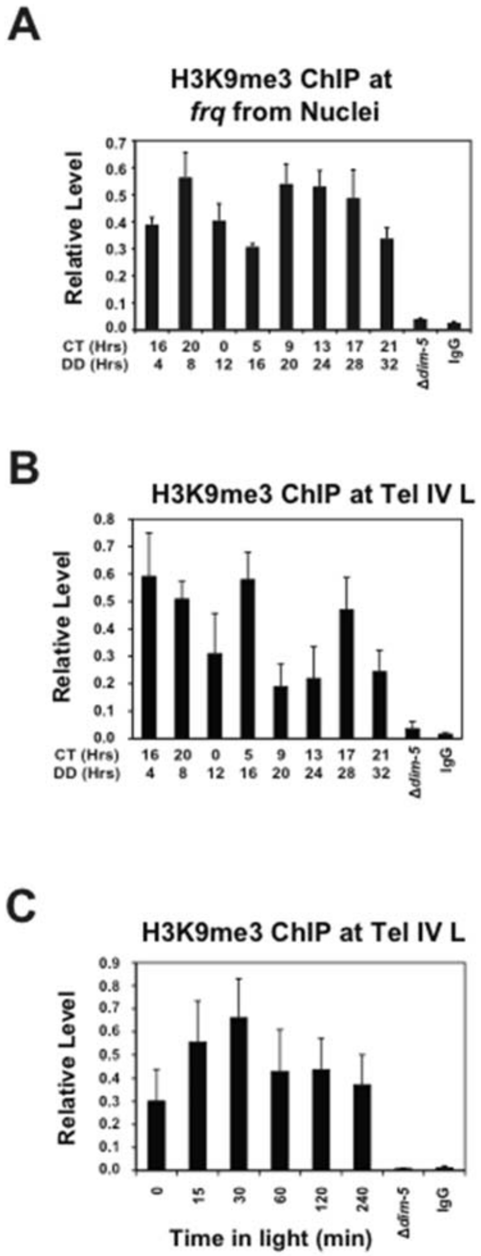

Figure S2 Supplemental H3K9me3 ChIP data. (A) Additional H3K9me3 ChIP experiments were performed at *frq* using isolated nuclei in an attempt to limit the background and cross-reactivity. (B) ChIP analysis of DNA from WT over circadian time examining the presence of H3K9me3 at Telomere IVL. (C) Same as A except examined in response to light.

Figure S3

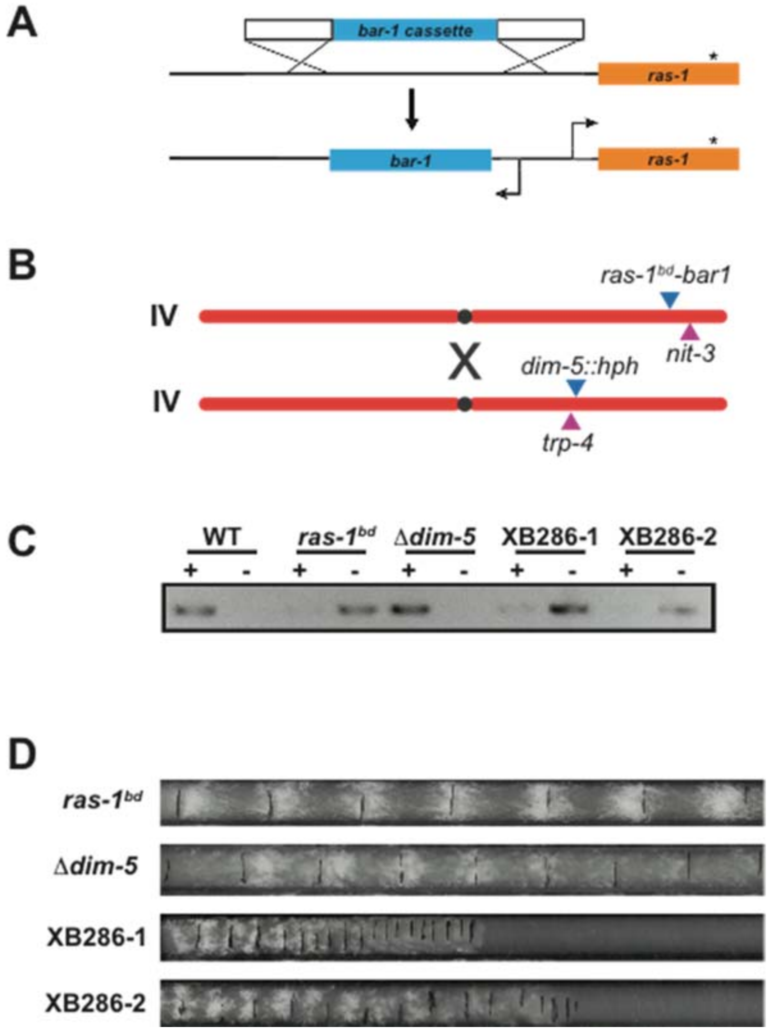

Figure S3 Synthetic affect between *Δdim-5* and *ras-1*^{bd}. The physical distance separating *dim-5* and *ras-1* is 1.64 megabases (Mb). The estimate of genetic distance between *dim-5* and *ras-1* is based on proximity and separation to previously identified genetic markers on right arm of Linkage Group IV. *trp-4* and *nit-3* are 1.8Mb apart and the genetic distance between these two loci range from 40 to 46 cM, indicating almost independent segregation. Despite this almost independent linkage, we were unable to isolate a spore containing both *dim-5::hph* and *ras-1*^{bd} from a standard cross examining 80 separate *dim-5::hph* spores. Therefore, we proceeded with a genetic selection depicted in this figure. (A) We placed the *bar-1* selectable marker upstream of *ras-1*^{bd}. In the design we specifically avoided disturbing the *ras-1* promoter. (B) This stain was crossed to *dim-5::hph* and we plated approximately 10,000 spores (determined using a hemocytometer) on plates containing both hygromycin and ignite. Of the approximately 10,000 spores plated, only 2 viable spores containing both *dim-5::hph* and *ras-1*^{bd}*-bar-1* were obtained. Based on the genetic linkages stated above, one would have expected greater than 1000-2000 spores. We then proceeded to confirm the presence of the *ras-1*^{bd} allele molecular methods. (C) The gel depicts the confirmation of the *ras-1*^{bd} single nucleotide polymorphism using SNAPPER. The + and - represent PCR reactions with wild-type and *ras-1*^{bd} specific primers respectively. The reactions with WT (FGSC2489), *ras-1*^{bd} and *Δdim-5* are controls and yielded expected products. The two progeny from a cross between *ras-1*^{bd} and *Δdim-5* (XB268-1 and XB268-2) were found to be positive for the *ras-1*^{bd} allele. (D) Representative race tubes from *ras-1*^{bd} *Δdim-5* and progeny XB-286-1 and XB286-2. Note the highly reduced growth is indicative of a synthetic effect when the two mutations are combined. There is also appears to be a gradual loss in growth and dampening of circadian output.

Figure S4.

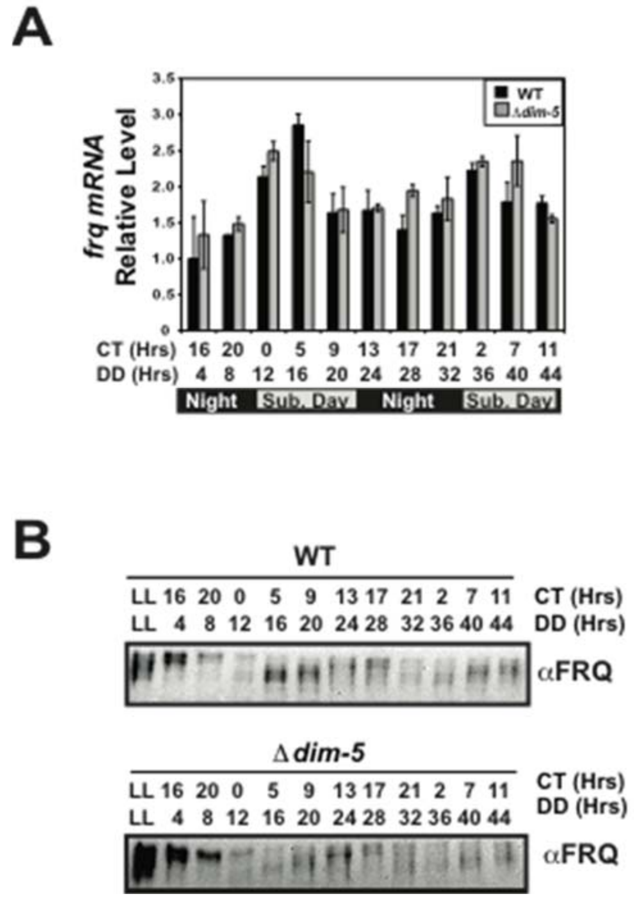

Figure S4 Molecular Rhythms in *Δdim-5*. (A) Molecular oscillations of the *frq* transcript were examined by RT-PCR in WT (FGSC2489) and *Δdim-5* (XB18-11) strains grown under circadian conditions. (B) Oscillations in FRQ were monitored by Western blot using an antibody specific to FRQ. The time in the dark (DD) and corresponding circadian time (CT) is indicated. Samples labeled LL indicates the strains were grown in constant light for 48 hrs.

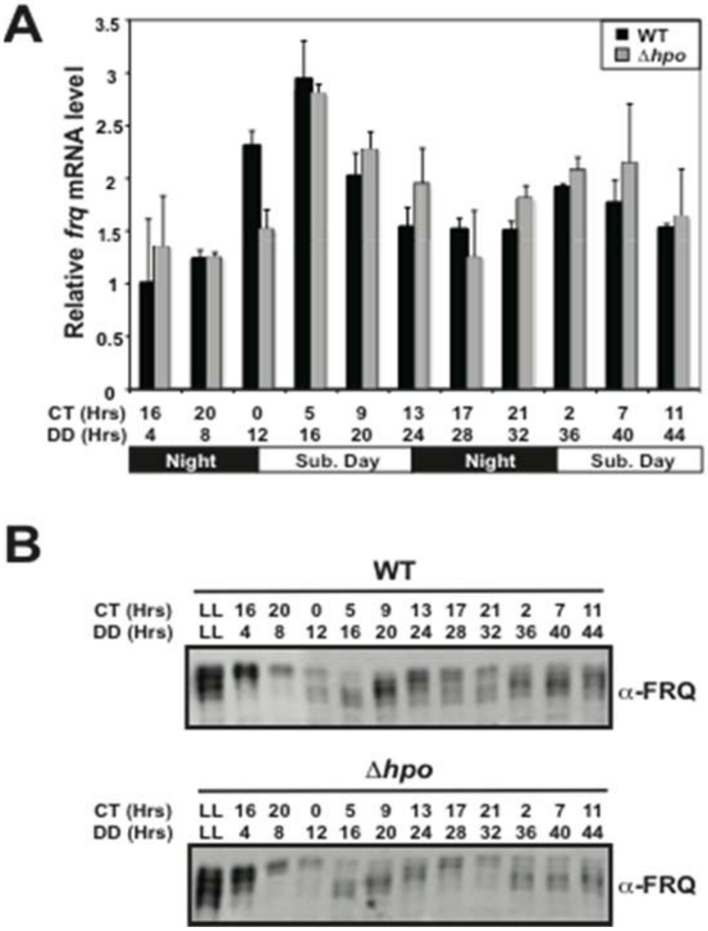

Figure S5 Molecular Rhythms in *hpo*. (A) Molecular oscillations of the *frq* transcript were examined by RT-PCR in WT (FGSC2489) and *hpo* strains grown under circadian conditions. (B) Oscillations in FRQ were monitored by Western blot using an antibody specific to FRQ. The time in the dark (DD) and corresponding circadian time (CT) is indicated. Samples labeled LL indicates the strains were grown in constant light for 48 hrs.

Figure S6.

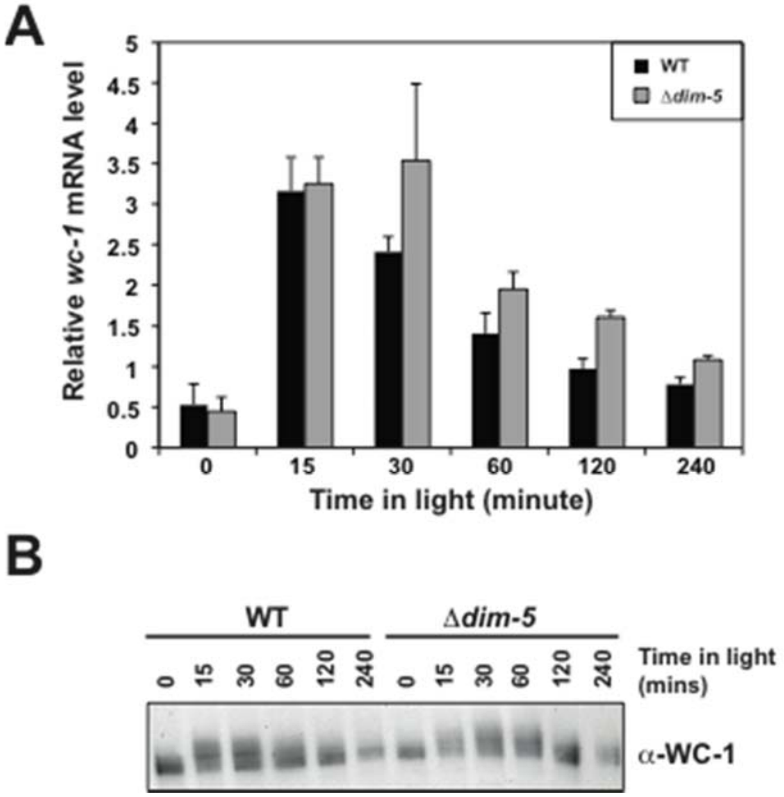

Figure S6 DIM-5 is not required for *wc-1* expression. (A) WT (FGSC2489) and *Δdim-5* (XB18-11) strains were grown in the dark then transferred to saturating light and harvested at the indicated time. RNA was extracted and transcript abundance was measured by RT-PCR with oligonucleotides specific for *wc-1.* (B) Total soluble protein was extracted from the samples in (A) then blotted for WC-1.
