# Supplementary material for: The Histone H3 Lysine 9 Methyltransferase DIM-5 Modifies Chromatin at frequency and Represses Light-Activated Gene Expression
Source: G3 (Bethesda). 2014 Nov 25;5(1):93–101. doi: 10.1534/g3.114.015446 (PMC4291474; doi:10.1534/g3.114.015446)
Supplement: Supporting Information [file supp_g3.114.015446_TableS1.pdf]

Table S1

Strain Table

Strain	Genotype	Source
FGSC2489	OR74, A (WT)	FGSC
FGSC11124	*wc-2::hph*, a	FGSC
FGSC15885	[*dim-5::hph, a*]	FGSC
XB18-4	*dim-5::hph*	This Study
XB18-5	*dim-5::hph*	This Study
XB18-11	*dim-5::hph*	This Study
XB18-13	*dim-5::hph*	This Study
90-2	*mus51::bar, a*	Colot *et al.*, 2006
T101	[*dim-2::nat1; mus51::bar, a*]	This Study
XB100-9	*chd-1::hph, A*	This study
XB104-2	*dim-2::nat1; chd-1::hph*	This Study
XB131-6	*chd-1::hph*, a	Belden *et al.*, 2011
XB136-6	*ras-1*^{bd}, A	Raduwan *et al.*, 2013
XB142-8	*dim-5::hph*, A	This Study
XL94-10	*frq-luc::bar, a*	Larrondo *et al.*, 2012
XB151-2	*dim-5::hph; frq-luc::bar*	This Study
XB230-9	*dim-5::hph; chd-1::hph*	This Study
XB230-12	*dim-5::hph; chd-1::hph*	This Study

* FGSC denotes Fungal Genetics Stock Center
