# Supplementary material for: The Histone H3 Lysine 9 Methyltransferase DIM-5 Modifies Chromatin at frequency and Represses Light-Activated Gene Expression
Source: G3 (Bethesda). 2014 Nov 25;5(1):93–101. doi: 10.1534/g3.114.015446 (PMC4291474; doi:10.1534/g3.114.015446)
Supplement: Supporting Information [file supp_g3.114.015446_FigureS1.pdf]

Figure S1.

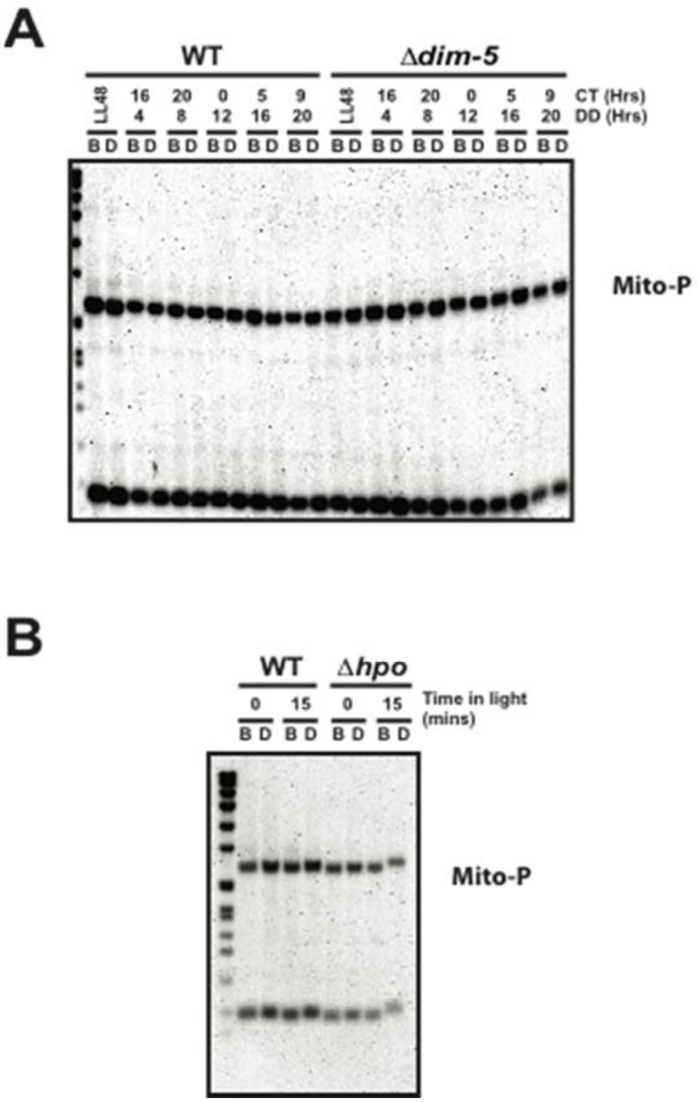

Figure S1 Control for DNA methylation in *Δdim-5* and *hpo*. (A) Methylation sensitive Southern blot from Figure 1 was stripped and re-probed with a probe that hybridizes to a region within the mitochondria genome. The figure indicates complete digestion in WT (FGSC2489) and *Δdim-5* (XB18-11). Time in the dark (DD) and corresponding circadian time (CT) is indicated. The restriction enzymes are the methyl-sensitive *Bfu*CI (B) and insensitive *Dpn*II (D). (B) Same as in A except the membrane was from Figure 1B that examined the *hpo* strain.
