# Supplementary material for: The Histone H3 Lysine 9 Methyltransferase DIM-5 Modifies Chromatin at frequency and Represses Light-Activated Gene Expression
Source: G3 (Bethesda). 2014 Nov 25;5(1):93–101. doi: 10.1534/g3.114.015446 (PMC4291474; doi:10.1534/g3.114.015446)
Supplement: Supporting Information [file supp_g3.114.015446_FigureS2.pdf]

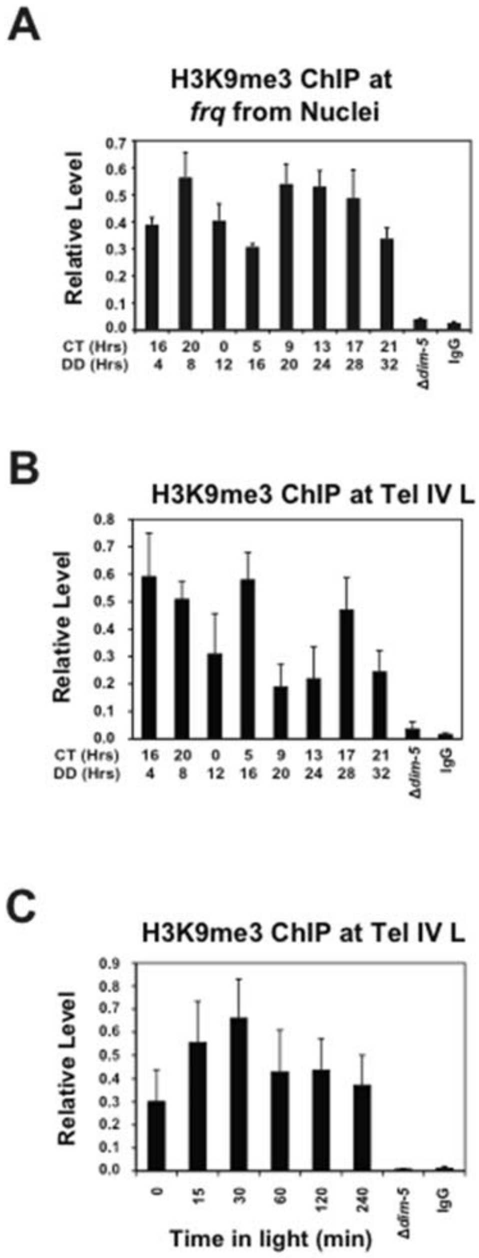

Figure S2 Supplemental H3K9me3 ChIP data. (A) Additional H3K9me3 ChIP experiments were performed at *frq* using isolated nuclei in an attempt to limit the background and cross-reactivity. (B) ChIP analysis of DNA from WT over circadian time examining the presence of H3K9me3 at Telomere IVL. (C) Same as A except examined in response to light.
