# Supplementary material for: The Histone H3 Lysine 9 Methyltransferase DIM-5 Modifies Chromatin at frequency and Represses Light-Activated Gene Expression
Source: G3 (Bethesda). 2014 Nov 25;5(1):93–101. doi: 10.1534/g3.114.015446 (PMC4291474; doi:10.1534/g3.114.015446)
Supplement: Supporting Information [file supp_g3.114.015446_FigureS3.pdf]

Figure S3

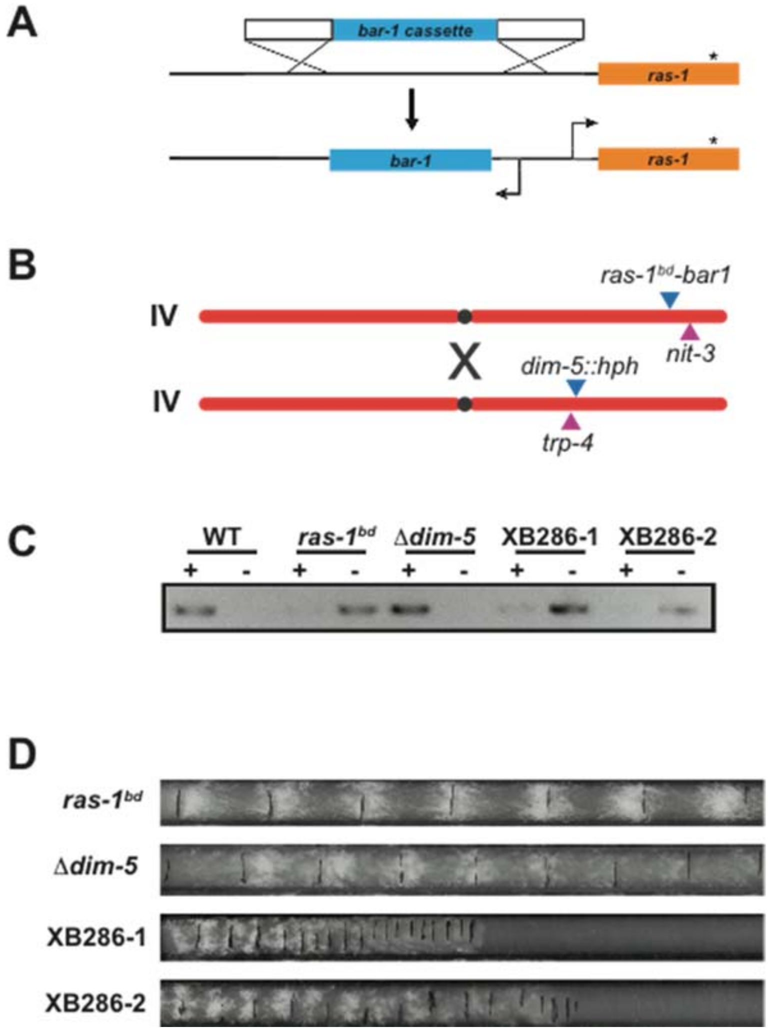

Figure S3 Synthetic affect between *Δdim-5* and *ras-1^{bd}*. The physical distance separating *dim-5* and *ras-1* is 1.64 megabases (Mb). The estimate of genetic distance between *dim-5* and *ras-1* is based on proximity and separation to previously identified genetic markers on right arm of Linkage Group IV. *trp-4* and *nit-3* are 1.8Mb apart and the genetic distance between these two loci range from 40 to 46 cM, indicating almost independent segregation. Despite this almost independent linkage, we were unable to isolate a spore containing both *dim-5::hph* and *ras-1^{bd}* from a standard cross examining 80 separate *dim-5::hph* spores. Therefore, we proceeded with a genetic selection depicted in this figure. (A) We placed the *bar-1* selectable marker upstream of *ras-1^{bd}*. In the design we specifically avoided disturbing the *ras-1* promoter. (B) This stain was crossed to *dim-5::hph* and we plated approximately 10,000 spores (determined using a hemocytometer) on plates containing both hygromycin and ignite. Of the approximately 10,000 spores plated, only 2 viable spores containing both *dim-5::hph* and *ras-1^{bd}-bar-1* were obtained. Based on the genetic linkages stated above, one would have expected greater than 1000-2000 spores. We then proceeded to confirm the presence of the *ras-1^{bd}* allele molecular methods. (C) The gel depicts the confirmation of the *ras-1^{bd}* single nucleotide polymorphism using SNAPPER. The + and - represent PCR reactions with wild-type and *ras-1^{bd}* specific primers respectively. The reactions with WT (FGSC2489), *ras-1^{bd}* and *Δdim-5* are controls and yielded expected products. The two progeny from a cross between *ras-1^{bd}* and *Δdim-5* (XB268-1 and XB268-2) were found to be positive for the *ras-1^{bd}* allele. (D) Representative race tubes from *ras-1^{bd} Δdim-5* and progeny XB-286-1 and XB286-2. Note the highly reduced growth is indicative of a synthetic effect when the two mutations are combined. There is also appears to be a gradual loss in growth and dampening of circadian output.
