# Supplementary material for: The Histone H3 Lysine 9 Methyltransferase DIM-5 Modifies Chromatin at frequency and Represses Light-Activated Gene Expression
Source: G3 (Bethesda). 2014 Nov 25;5(1):93–101. doi: 10.1534/g3.114.015446 (PMC4291474; doi:10.1534/g3.114.015446)
Supplement: Supporting Information [file supp_g3.114.015446_FigureS5.pdf]

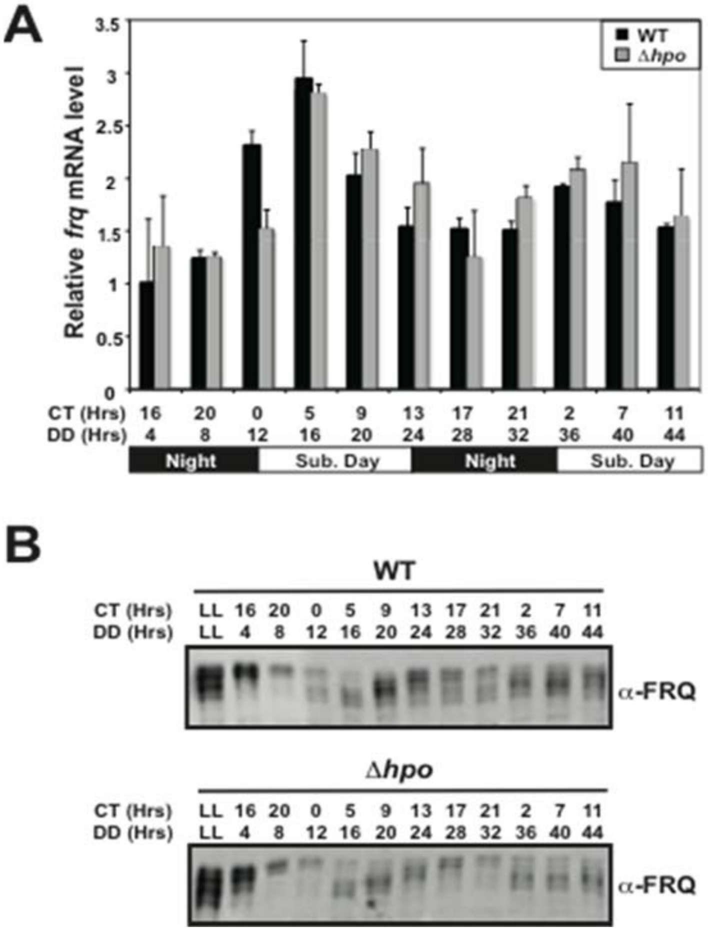

Figure S5 Molecular Rhythms in *hpo*. (A) Molecular oscillations of the *frq* transcript were examined by RT-PCR in WT (FGSC2489) and *hpo* strains grown under circadian conditions. (B) Oscillations in FRQ were monitored by Western blot using an antibody specific to FRQ. The time in the dark (DD) and corresponding circadian time (CT) is indicated. Samples labeled LL indicates the strains were grown in constant light for 48 hrs.
