# Supplementary material for: The Histone H3 Lysine 9 Methyltransferase DIM-5 Modifies Chromatin at frequency and Represses Light-Activated Gene Expression
Source: G3 (Bethesda). 2014 Nov 25;5(1):93–101. doi: 10.1534/g3.114.015446 (PMC4291474; doi:10.1534/g3.114.015446)
Supplement: Supporting Information [file supp_g3.114.015446_FigureS6.pdf]

Figure S6.

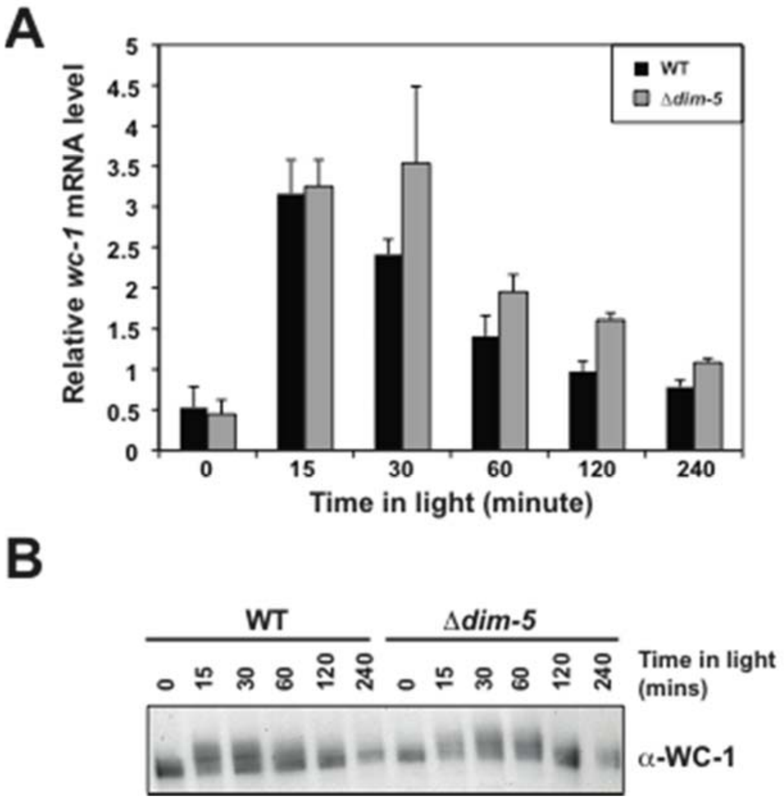

Figure S6 DIM-5 is not required for *wc-1* expression. (A) WT (FGSC2489) and *Δdim-5* (XB18-11) strains were grown in the dark then transferred to saturating light and harvested at the indicated time. RNA was extracted and transcript abundance was measured by RT-PCR with oligonucleotides specific for *wc-1.* (B) Total soluble protein was extracted from the samples in (A) then blotted for WC-1.
